# What imaging does my AIS patient need? A multi-group survey of provider preferences

**DOI:** 10.1007/s43390-024-00995-9

**Published:** 2024-11-04

**Authors:** Jenny L. Zheng, Ying Li, Grant Hogue, Megan Johnson, Jason B. Anari, Maia D. Regan, Keith D. Baldwin

**Affiliations:** 1 Division of Orthopaedic Surgery, Children’s Hospital of Philadelphia, Perelman School of Medicine at the University of Pennsylvania, Philadelphia, PA USA; 2 Department of Orthopaedic Surgery, C.S. Mott Children’s Hospital, University of Michigan Health, Ann Arbor, MI USA; 3 Orthopedic Center, Boston Children’s Hospital, Boston, MA USA; 4 Texas Scottish Rite Hospital for Children, Dallas, TX USA

**Keywords:** Adolescent idiopathic scoliosis, Imaging, Survey, Radiography, Nonoperative

## Abstract

**Introduction:**

Adolescent idiopathic scoliosis (AIS) is a common diagnosis managed by pediatric orthopedic surgeons with nonoperative radiographic monitoring representing a cornerstone of treatment. Differences in practices and techniques for obtaining radiographic studies contribute to variation, cost of care, and hamper data aggregation. We surveyed several large organizations dedicated to children’s orthopedics or scoliosis care to obtain a consensus for radiographic evaluation of AIS.

**Methods:**

A REDCap-based survey was developed across four institutions and beta-tested by staff and fellows from a single institution. The finalized survey was distributed to members of POSNA, PSSG, and SOSORT, and shared on social media. Participants were asked to rank the importance of various datapoints in radiographic assessment of the spinal deformity, skeletal maturity, and study indications during initial, subsequent, preoperative, and final office visits for AIS. Response rate for the overall group was 26%.

**Results:**

Cobb angle was considered the most important (> 94%) radiographic index across all time points. For positioning, 46% of respondents favored arms bent touching clavicles as the ideal positioning for X-rays, and another 24% favored arms down with palms forward (Table 2). The majority of respondents obtain lateral X-rays at the first visit (99%) and at the preoperative visit (70%). At the preoperative visit, sagittal contour (86%), apex location (85%), and Lenke classification (73%) were considered important factors to record. Flexibility studies are primarily obtained at the preoperative visit (89%) and 81% of respondents prefer bending films as the flexibility technique of choice. Regarding measures of skeletal maturity, Sanders bone age was considered to be the most important by over 70% of respondents across initial, subsequent, preoperative and brace wean visits (Fig. 2). MRIs were obtained routinely by 34% of respondents and only when the patient had a concerning symptom or finding for 67% of respondents.

**Conclusions:**

Despite large variations in radiographic examination of AIS, large areas of agreement were found. It is important to establish standards for positioning patients, evaluating skeletal maturity, and obtaining assessments including lateral views, flexibility studies, and advanced imaging. Establishing common practices for radiographic evaluation of AIS will allow for less variation in care and for critical questions to be answered through registry formation and large multicenter data collection.

**Significance:**

This study establishes current practitioner opinion on the radiographic evaluation of the AIS patient. Minimum data sets are useful for data aggregation and answering research questions in the face of data variability.

**Level of evidence:**

Level V.

## Introduction

Adolescent idiopathic scoliosis (AIS) affects up to 5.2% of at-risk children globally and is one of the most common spinal conditions managed by pediatric orthopedic surgeons [1–4]. Routine radiographic monitoring is a critical component of nonoperative AIS diagnosis and clinical care, and the gold standard for diagnosing and monitoring scoliosis has been routine surveillance with standing anteroposterior (AP) or posteroanterior (PA) scoliosis X-ray films [5–7].

Despite advancements in imaging techniques, there are still significant differences in practices and techniques for obtaining radiographic studies and no consensus regarding standard practice for radiographic assessment of AIS [8–11]. A previous systematic review by the United States Preventive Services Taskforce (USPSTF) on scoliosis screening was unable to perform meta-analyses of results due to the heterogeneity of data collected [12]. This variability not only contributes to higher cost of care but also hampers large-scale data aggregation [8, 13]. Large-scale registry study of AIS is necessary to elucidate optimal decisions for observation, bracing, or surgical intervention. Variation in data collection adds extra statistical noise to a topic which already is plagued by variability in presentation and progression. A lack of consensus regarding an optimal standardized radiographic exam renders registry studies difficult to aggregate properly. Thus, it is critical to establish common data elements for radiographic evaluation of AIS as previously produced for other diagnoses including sports-related concussions and spinal cord injuries [14, 15].

The purpose of this study was to determine an expert consensus of important radiographic data elements for evaluation and treatment of AIS through a cross-sectional survey of the membership of multiple large organizations of physicians and allied health professionals who treat scoliosis.

## Methods

### Survey development

A REDCap questionnaire was developed across four institutions involved in the Scoliosis Non-Operative REgistry Study (SNORES) Group, a collaboration developed with funding from the Scoliosis Research Society and dedicated to the study and improvement of nonoperative treatment of adolescent idiopathic scoliosis. The questionnaire aimed to evaluate various aspects of clinical AIS visits including patient histories, physical examination measurements, radiographic examination practices, and bracing at a patient’s new patient visit, subsequent visits, immediate preoperative visit, and final/brace wean visit. Survey questions asked respondents to either rate the importance of data elements as very to not important or rank elements from most to least important.

The survey structure was divided into the following sections: (1) respondent demographics, (2) patient history, (3) physical examination, (4) radiographic examination, (5) other elements of visits including follow-up frequency, MRI recommendations, and bracing practices. Adaptive branching questions were utilized to better elucidate provider preferences. An optional text field was included at the end of the survey for respondents to recommend additional data elements that were not included in the survey. The longest version of the survey contained 132 total fields and required less than 10 min to complete.

Survey beta-testing was performed by two attending orthopedic surgeons and four pediatric orthopedic surgery clinical fellows from a single institution. Survey question wording and content was improved based on feedback from the beta testers. Institutional Review Board (IRB) approval was obtained from our local institution.

### Survey distribution

The questionnaire was distributed via REDCap to 1400 members of the Pediatric Orthopaedic Society of North America (POSNA), 180 members of the Pediatric Spine Study Group (PSSG), and 224 members of the International Society on Scoliosis Orthopaedic and Rehabilitation Treatment (SOSORT). The survey was reviewed and approved for distribution to the POSNA membership by the POSNA Evidence-Based Practice Committee, to the PSSG membership by the Executive Director, and to the SOSORT membership by the SOSORT Scientific Committee.

POSNA, PSSG, and SOSORT members were invited via email to participate in the survey. The survey link was also shared on Twitter by members of the research team. A short description of the survey was provided prior to the opt-in questions. Respondents could opt-in if they had experience managing AIS and were willing to voluntarily complete the survey. Informed consent was obtained by successfully answering the opt-in questions. Anonymous responses were collected over a 5-month period.

### Analysis

Survey responses with at least the first three sections completed were included in our final analysis. Results from the survey portions surrounding radiographic evaluation and care were evaluated in this paper. Standard descriptive statistics were used evaluate survey responses including frequencies of responses. Based on the number of POSNA members who self-identify as spine specialists (280), the total number of PSSG members (180), and the total number of SOSORT members (224) at the time of survey distribution, we estimated an overall response rate of 26%.

Recommendations for a minimum data set were established based on majority agreement on the importance of evaluated data elements. Data elements were defined as critical if a supermajority of 60% of respondents perceived them as important. The 60% threshold, based on a binominal distribution of all evaluated responses, provides a 99% confidence interval that the normal bound of favorable responses is greater than 50%.

### Results

A total of 181 responses were reviewed (26% response rate) from the United States and 22 other countries (Table 1). An additional 79 survey responses were received but excluded from analysis due to lack of completion (68% completion rate). Of respondents, 86% were physicians and 14% were allied health professionals. 80% of respondents worked at a free-standing pediatric hospital or in a pediatric space within an adult hospital, and 82% were academic, with the majority (57%) seeing 150 + unique AIS patients per year. Only seven individuals (3.9%) responded that they see 0–25 patients annually. Of these respondents, years in practice ranged from 0–10 up to 30 + . On subgroup analysis of results by physicians alone, there were no large discrepancies within the overall trends of preferences.

**Table 1:** Respondent demographics

*N* = 181	*n*	%
*Role*		
Physician	156	86.2
Physical therapist	16	8.8
Orthotist	6	3.3
Chiropractor	3	1.7
Type of hospital		
Free standing pediatric hospital	111	61.3
Pediatric practice within space inside adult hospital	34	18.8
Community/general hospital	10	5.5
Other	26	14.4
Type of practice		
Academic	149	82.3
Non-academic	30	16.6
Years in practice		
0–10	57	31.5
11–20	52	28.7
21–30	32	17.7
30 +	37	20.4
AIS patients seen annually		
0–25	7	3.9
26–50	17	9.4
51–100	31	17.1
101–150	23	12.7
151 +	103	56.9
Geographic location		
United States	129	71.3
Northeast	33	18.2
Midwest	20	11.0
West	23	12.7
South	30	16.6
Puerto Rico	2	1.1
Canada	6	3.3
Europe	27	14.9
Asia	8	4.4
South America	3	1.7
Australia	3	1.7

**Fig. 1:**
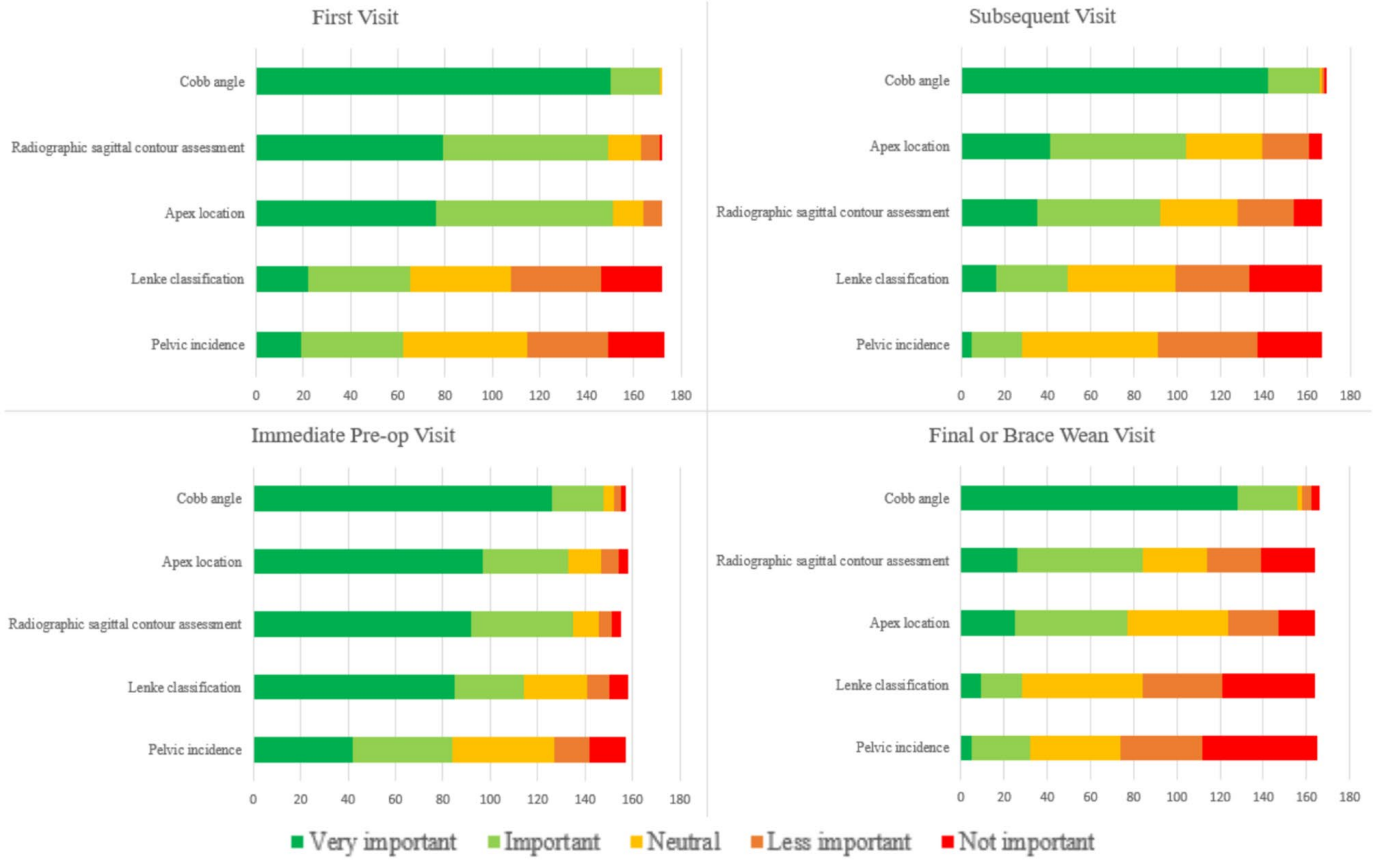
Respondent rankings of the importance of radiographic data elements at each visit type

Cobb angle was considered the most important (> 94%) radiographic index across all time points (Fig. 1). When considering ideal positioning for AP radiographs, 46% of respondents favored arms bent touching clavicles as the ideal positioning for X-rays, and another 24% favored arms down with palms forward (Table 2). The majority of respondents obtain lateral X-rays at the first visit (99%) and at the preoperative visit (70%).

**Table 2:** Respondent preferences when obtaining additional radiographic studies

	*n*	%
Ideal position for radiographs		
Arms bent touching clavicle	75	46.0%
Arms down with palms forward	39	23.9%
Arms positioned with proximal humerus in AP	20	12.3%
Hands over head	14	8.6%
Arms straight out forward	8	4.9%
Other*	7	4.3%
When do you obtain a lateral view?		
First visit	161	98.8%
Subsequent visits	39	23.9%
First in-brace visit	49	30.1%
Pre-op visit	114	69.9%
Final or brace wean visit	69	42.3%
When do you obtain flexibility studies?		
First visit	19	11.7%
Subsequent visits	5	3.1%
First in-brace visit	7	4.3%
Pre-op visit	145	89.0%
Final or brace wean visit	10	6.1%
What flexibility study do you use most commonly?		
Bending film	125	80.6%
Traction bending film	15	9.7%
Bolster bending film	7	4.5%
Traction only film	8	5.2%

*Other: Arms down with palm towards hip, hand on wall at level of clavicle, hands on EOS wall next to head, hands up by face

At the patient’s first visit, major curve apex location (88%) and sagittal contour assessment (87%) were also considered important by the majority of respondents. At subsequent visits, Cobb angle and apex location (62%) remained of critical importance while sagittal contour decreased slightly in importance (87% down to 55%). At the preoperative visit, sagittal contour (87%), apex location (84%), and Lenke classification (72%) were all considered important factors to record. Pelvic incidence did not reach the 60% threshold to be considered a critical data element across all visit types.

Flexibility studies are primarily obtained at the preoperative visit by 89% of respondents, with bending films as the preferred flexibility technique by 81% of participants.

**Fig. 2:**
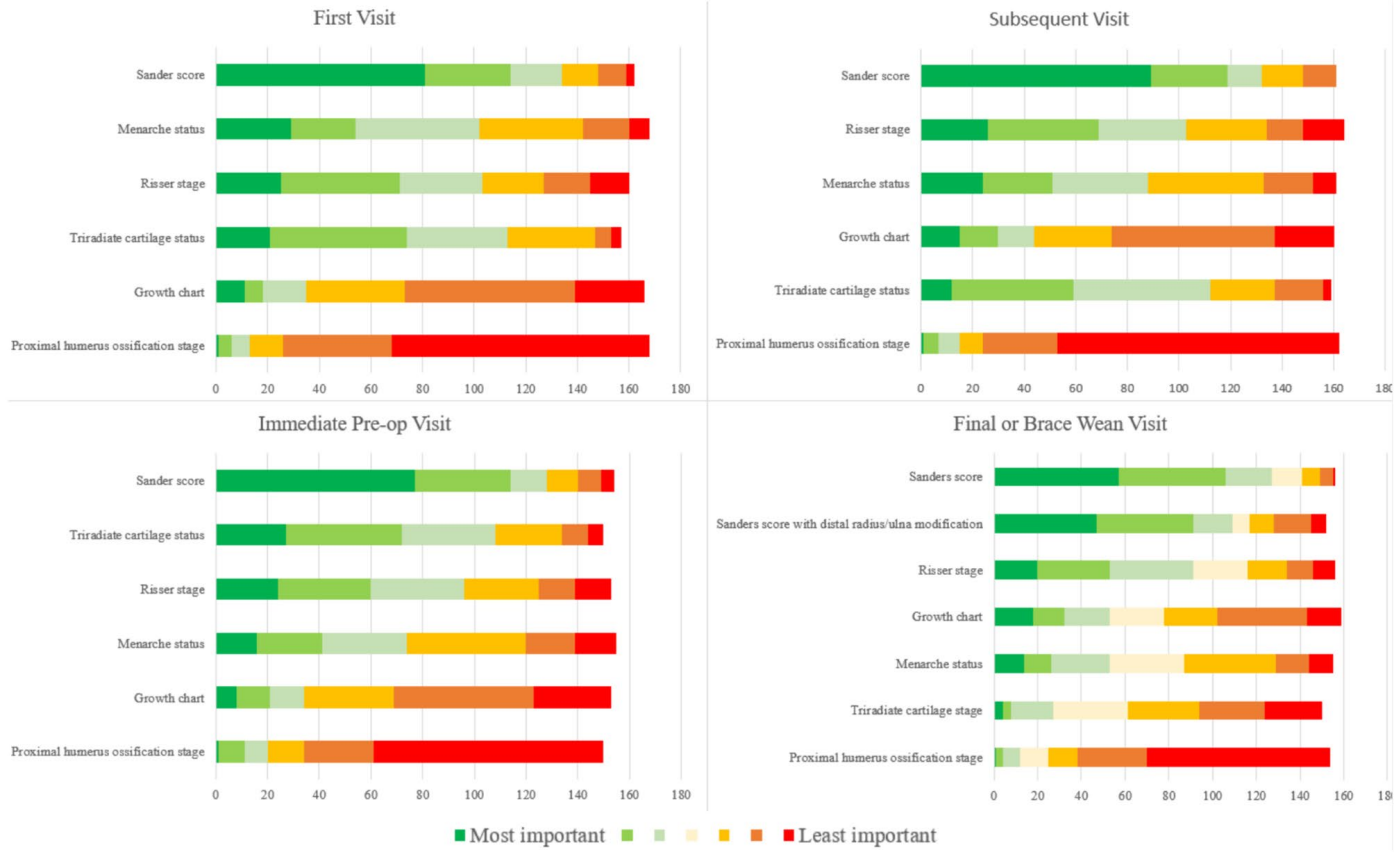
Respondent rankings of importance of skeletal maturity measurements at each visit type

Regarding measures of skeletal maturity, Sanders bone age was considered to be the most important by over 70% of respondents across initial, subsequent, preoperative and brace wean visits (Fig. 2). Menarche status, triradiate cartilage status, and Risser stage were also considered important (> 60%) during the patient’s first radiographic evaluation. At subsequent visits, menarche status becomes slightly less important (60% to 55%) while triradiate cartilage status and Risser sign remained important (> 60%). These preferences remained consistent at the immediate preoperative visit.

MRIs were obtained routinely by 34% of respondents and only when the patient had a concerning symptom or finding by 67% of respondents. 

## Discussion

The vast majority of children with adolescent idiopathic scoliosis will only require nonoperative care, including routine radiographic surveillance of their scoliotic curve progression [1–5, 7]. Despite significant advancements in imaging techniques, there is no consensus regarding critical radiographic data elements and practices [8–11]. In particular, there are no standard methods for predicting changes in skeletal maturity, peak growth velocity, and curve progression [11]. Establishing common data elements in radiographic assessment of AIS is necessary in order to design large-scale registry studies. Such studies may ultimately decrease the heterogeneity of care in AIS and decrease the need for unnecessary imaging and radiation exposure [13]. This study aimed to report an expert consensus on critical radiographic data elements in AIS care among members of POSNA, PSSG, and SOSORT across a patient’s first, subsequent, immediate preoperative, and final/brace visits.

Here, we propose an 11-item minimum data set for the radiographic evaluation of AIS (Table 3). This data set includes recommendations for assessing deformity severity, skeletal maturity, and imaging views. The goal of this study was to establish a treatment template that is inclusive of AIS patients at large. Still, physicians should exercise their clinical judgment when treating individual patients. Moreover, while there is a general consensus regarding the importance of the radiographic data elements presented here, this study also highlights the enormous variability that exists within radiographic practices in the management of AIS which renders large-scale study of nonoperative care so difficult. The variability presented in these results serves as a call to establish more standardized radiographic practices.

**Table 3:** Minimum data set recommendations for radiographic assessment of AIS

	First visit	Subsequent visits	Preoperative visit	Final/brace wean visit
Deformity measurements				
1. Cobb angle	X	X	X	X
2. Apex location	X	X	X	
3. Sagittal contour	X		X	
4. Lenke classification			X	
Skeletal maturity measurements				
5. Sanders	X	X	X	X*
6. Triradiate cartilage	X	X	X	
7. Risser stage	X	X		
8. Menarche status	X	X		
Additional views				
9. Lateral view	X		X	
10. Bending film			X	
11. MRI			If indicated	

*with distal radius/ulna modifications

### Radiographic measurements

Across all visits, major curve Cobb angle and apex location should be documented. The vast majority survey respondents (> 94%) recommend close attention to the progression of Cobb angle across visits. Attention to Cobb angle is critical for adequate nonoperative AIS care and decisions to observe, brace, or operate. Of adolescents diagnosed with AIS, only 10% of curves will require any orthopedic intervention [16]. Natural history studies suggest bracing for patients with 25–40 degree curve magnitudes and consideration of surgical intervention when curve magnitudes progress to 50 degrees [17, 18]. Apex location was also perceived as important across all visit types by the majority (> 60%) of respondents. This finding is supported by previous studies that suggest curves with higher apex levels have higher risk of progression [19, 20]. A study from Strube et al. also found asymmetric major curves with a lower apex to be associated with lower success rates of nonoperative treatment [21]. Careful documentation of major Cobb angle and location is, therefore, important for monitoring curve progression.

At a patient’s first visit, physicians should additionally evaluate radiographic sagittal contour. AIS is a complex 3-dimensional deformity involving the coronal, axial, and sagittal plane. A previous study from Schlosser et al. found 49% of mild thoracic AIS curves (10–20 degrees) and 63% of severe AIS curves (> 45 degrees) to present with thoracic hypokyphosis [22]. As such, it is important to assess radiographic sagittal alignment when patients present for evaluation of AIS and during preoperative assessment.

At the immediate preoperative visit, sagittal contour and Lenke classification should be documented. Lenke classification has been demonstrated to improve surgical planning and reduce variation in treatment approaches, although other clinical and radiographic characteristics of a patient’s unique deformity beyond Lenke classification should still be considered during surgical decision-making [23, 24]. Still, further research is necessary to evaluate the effects of adherence to this classification system [24]. In addition, 53% of survey respondents recommended evaluation of pelvic incidence at the patient’s immediate preoperative radiographic exam. Although this result did not meet the 60% threshold for critical importance, pelvic incidence may still be evaluated as an additional measure of sagittal balance, although perhaps with less weight than other radiographic measurements.

### Skeletal maturity

Sanders staging has grown in popularity and now appears to have surpassed Risser staging as the preferred method for assessing skeletal maturity. Sanders stage should be evaluated at every visit with the incorporation of distal radius and ulna classifications at the patient’s final/brace wean visit. Over 70% of survey respondents favor obtaining Sanders score at every visit. Sanders staging is reliable and correlates well with curve progression, although further study is necessary to generate a more robust understanding of the correlation between Sander bone age stage, curve size, and risk of curve progression [25, 26]. Routine documentation of Sanders stage will help future large-scale registry studies better predict progression of AIS. Previous studies also recommend the earliest point for brace weaning to be at Sanders Stage 8 and radius Grade 10 and/or ulna Grade 9 in order to decrease risk of curve progression after weaning [27]. Practitioners should pay particular attention to Sanders staging at the radius and ulna when deciding on brace discontinuation to maximize treatment efficacy.

Risser stage and menarche status are critical to evaluate at a patient’s first and follow-up AIS visits. Risser staging has been found to have lower sensitivity during peak growth velocity and lower interobserver reliability compared to the Sanders classification [28]. Thus, in accordance with previous studies, we recommend use of Sanders as the primary measure of maturity to guide treatment options. [28, 29]

Furthermore, because curve progression is closely associated with growth rate, we recommend close observation of menarche status. Over 55% of respondents recommend documenting menarche status at the first and follow-up visits. An estimated 75% of curves from 20 to 30 degrees at onset of puberty go on to require surgical intervention [16, 30, 31]. Nearly 100% of patients with curves over 30 degrees at the onset of puberty will require surgery [16, 30, 31]. These growth measurements serve as important data points when assessing risk of curve progression. Similarly, triradiate cartilage status should also be assessed at the first visit, subsequent visits, and preoperative visit. Closed triradiate cartilage is associated with both decreased risk of curve progression and decreased risk of loss of correction following spinal fusion [32, 33]. As such, it is important to closely monitor and document a patient’s skeletal maturity and growth velocity following these data elements.

### Positioning techniques and additional imaging

When obtaining radiographs, we recommend the arms bent touching clavicles method for positioning, which was favored by 46% of survey respondents. A study from Pasha et al. evaluated functional standing balance between three different arm positions (hands on clavicles, hands on wall, and hands hanging down) and found the greatest reliability in assessment of AIS using the clavicle position [34]. The clavicle positioning has also been suggested to best represent the patient’s functional balance on lateral radiographs [9]. This preference is likely in the setting of simultaneous acquisition of coronal and sagittal images and would likely not be as important when obtaining a coronal view alone. Our study highlights a marked lack of consensus on positioning techniques and a marked need to establish more uniform practices. Doing so will help decrease variability in care across health systems and in data aggregation for multicenter registry studies [34].

A lateral view should be obtained at the patient’s first visit and immediate preoperative visit. The majority of respondents reported obtaining lateral views at the first visit (99%) and the preoperative visit (70%), practices supported by the existing literature [35]. Given the particular interest in 3-dimensional deformity correction, particularly in patients with hypokyphotic deformities, we recommend preoperative lateral views for adequate assessment and correction of sagittal deformities [36, 37]. Recent developments in bracing research also suggest increased importance of routinely monitoring in-brace sagittal correction [38]. It is possible that lateral views may become more important as more bracing data becomes available.

Flexibility studies should be obtained at the preoperative visit with side bending as the preferred technique. 89% of survey respondents reported obtaining flexibility studies at the preoperative visit with 81% using bending films. Previous studies have showed benefits of different techniques such as side-bending, bolster/fulcrum-bending, traction, and push-traction bending, although side-bending films are generally considered the standard method for assessing flexibility of idiopathic curves less than 60 degrees [39–43]. Given the considerable variability in practice patterns, it is critical to establish a consensus on the best technique to use when evaluating preoperative curve flexibility. Curve flexibility may also be assessed before initiation of bracing, although our study did not assess the popularity of this preference.

Practices for obtaining preoperative MRIs are widely variable. 67% of survey respondents reported obtaining preoperative MRIs for patients with concerning histories, physical exams, or radiographic presentations, while the remaining 33% report obtaining preoperative MRIs for every patient by routine. Current literature recommends advanced imaging in the presence of atypical findings [44–46]. Other studies suggest up to 1 in 7 patients with normal presentations demonstrate neuroaxial disease on MRI and thus recommend MRI for all patients regardless of neurological examination findings [47, 48]. A prior meta-analysis of 4746 patients found an overall 8% of patients with AIS to have neuraxial abnormalities, 33% of whom went on to experience changes to their surgical plan [49]. Although the majority of clinicians continue to prefer selective use of MRI, the additional diagnostic value of routine advanced imaging should not be underestimated. Further study is needed to establish a consensus for MRI screening strategies. Accordingly, physicians should exercise careful attention to any atypical presentations and consider the threshold degree of uncertainty they are willing to accept when determining whether advanced imaging is appropriate. All together, these studies point to the need for more uniform large-scale study of AIS as curves progress near surgical range.

### Limitations

This study is not without its limitations. In order to produce a concise survey to decrease redundancies and survey fatigue, available answer choices did not encompass every element and technique used for radiographic AIS evaluation. In particular, this study did not evaluate radiographic practices during bracing treatment, such as frequency of in-brace/out-of-brace X-rays and time spent out of the brace prior to out-of-brace X-rays. These items were omitted to reduce likelihood of respondent fatigue. Survey response options also did not include all measures of skeletal maturity, such as proximal femur and olecranon ossification stages. Furthermore, weighted analysis of reported preferences based on patient volume may have provided a more accurate assessment of the care that the average patient is likely to receive. However, the goal of this study was to understand practice patterns of clinicians at large in order to establish standardized practice recommendations and a minimum data set to encompass the majority of patients and to decrease variability among current practices. As such, the authors felt that the level of detail of the questionnaire and analysis of results by number of respondents were appropriate in meeting the study goals.

In addition, the overall response rate for this survey estimated to be 26% of qualified members of POSNA, PSSG, and SOSORT. While survey participants represent a diverse demographic distribution, our results may not represent the true heterogeneity of practice patterns for AIS providers at large beyond these institutions. Due to the survey’s anonymous nature, it is also difficult to account for provider preferences tied with specific practice sites, although such associations may certainly exist. Furthermore, survey studies appear to have increased in popularity while also demonstrating decreasing responses rates. This trend may be due to growing survey fatigue. Previous studies report responses rates as a low as 15% among orthopedic surgeons [50]. While this survey study attempted to capture the practices of clinicians nationally and even internationally with nearly 30% of respondents from outside of the USA, our results may not fully represent the heterogeneity of preferences of AIS providers at large beyond POSNA, PSSG, and SOSORT membership. Still, the 60% threshold for critical importance in our study demonstrates with 99% confidence that the population at large would reach 50% agreement of the minimum data set elements presented in our results. These recommendations are further limited to the radiographic evaluation of patients with AIS and may not be translatable to patients with other etiologies of scoliosis.

Although there are large variations in radiographic examination of AIS, large areas of agreement were found. Here, we proposed a minimum data set of radiographic measurements, skeletal maturity markers, and recommendations for patient positioning and additional imaging. Clinicians should move toward standardizing radiographic practices and techniques to decrease large practice variability among institutions. The results of our study aim to establish common practices as the critical groundwork for future registry-based studies to improve both nonoperative treatment and surgical planning for AIS.

## Data Availability

Data included in this study may be available from the corresponding author upon reasonable request.
